# Psychological Safety for Mental Health in Elite Sport: A Theoretically Informed Model

**DOI:** 10.1007/s40279-023-01912-2

**Published:** 2023-09-22

**Authors:** Courtney C. Walton, Rosemary Purcell, Vita Pilkington, Kate Hall, Göran Kenttä, Stewart Vella, Simon M. Rice

**Affiliations:** 1 Melbourne School of Psychological Sciences, University of Melbourne, Melbourne, Australia; 2 Elite Sports and Mental Health, Orygen, Melbourne, Australia; 3 Centre for Youth Mental Health, The University of Melbourne, Melbourne, Australia; 4 Australian Football League, Melbourne, Australia; 5 School of Psychology, Deakin University, Geelong, Australia; 6 The Swedish School of Sport and Health Sciences, Stockholm, Sweden; 7 School of Human Kinetics, University of Ottawa, Ottawa, Canada; 8 Global Alliance for Mental Health and Sport, School of Psychology, University of Wollongong, Wollongong, Australia

## Abstract

Elite sports contexts are highly pressurised and frequently enforce a win-at-all-costs approach. This narrow focus on performance outcomes can potentially contribute in negative ways to the mental health of those within these environments. In this *Current Opinion* paper, we propose a model that outlines how key elements contributing to psychologically safe or unsafe environments may contribute to better or worse mental health outcomes, respectively. In an environment in which individuals feel safe to show their authentic selves rather than ‘wear a mask’, different experiences of mental health are likely to be normalised, help-seeking behaviour increased, and thus, mental health outcomes enhanced. We outline how sports teams and organisations can contribute to this through the creation of appropriate policies and procedures, in addition to leaders modelling and reinforcing positive cultural norms. It is intended that the theoretical model can inform stakeholders in elite sport as well as future research directions.

## Key Points

**Table:** 

Psychological safety has increasingly been linked to mental health in elite sports contexts. However, there has been very little discussion or evidence as to how this actually occurs.
We propose a theoretically informed model of how psychologically safe environments might lead to increased help-seeking and better mental health outcomes. Specifically, this may occur when individuals feel safe to show their authentic self, perceive their environment to support mental health needs, and psychological harms are minimised throughout the organisation or team.
This is the first conceptualisation of how psychological safety in elite sport may contribute to enhanced mental health outcomes. The model is testable, and further studies are now necessary to support or adapt the different processes we outline.

## Introduction

Elite and professional sports contexts are highly pressurised environments, frequently characterised by a narrow focus on performance outcomes. Unfortunately, this unrelenting demand for excellence can compromise health and well-being, with risks of abusive behaviour also present [1, 2]. It is now clear that athletes in elite sports experience mental ill-health at similar rates to the general population [3, 4], with coaches and support staff also increasingly recognised as at risk [5, 6]. It is critical that approaches to supporting mental health in elite sport move beyond the individual, to incorporate an understanding of the broader socioecological factors that contribute to mental health [2, 7, 8]. In addition to key structural contributors to stress and mental ill-health in elite sport (e.g. financial insecurity, performance pressures, etc.), the relational dynamics and cultures that exist within these contexts are pertinent to understanding the emergence of negative outcomes for individuals and organisations [9].

In parallel with the increasing awareness of mental health in elite sport, a growing body of research and practice has considered the concept of ‘psychological safety’ [10, 11]. Originating in the field of organisational psychology and primarily focussed on workplace effectiveness, psychological safety is now both variably defined and applied in sport. This inconsistency poses challenges to interpreting the current state of research, and any practical considerations that can be implemented within sports environments. While psychological safety has been broadly referred to in discussions about mental health in sport, limited direct evidence of this relationship exists [12–14].

While acknowledging the important influence of psychological safety on performance, here we focus specifically on how psychological safety may be critical within holistic socio-ecological approaches that support mental health in elite sport. Interestingly, recent changes to occupational health and safety international standards now include an emphasis on identification and minimisation of psycho-social risks in the workplace,^1^ and elite sport employers are not exempt. Thus, psychological safety may play a key role in achieving this.

In this *Current Opinion,* we outline how psychological safety has previously been conceptualised and summarise the unique aspects of elite sport which may contribute to mental ill-health. By elite sport, we refer to both elite and professional environments where performance pressures are at their highest [15]. These ideas may also apply to talent pathway and elite youth development programmes, although we have not explicitly discussed this here given the unique developmental considerations which must be considered in youth [16, 17]. We then provide a theoretically informed and empirically testable model outlining how core ingredients of psychological safety may contribute to mental health in this context. Thus, rather than introducing a new definition of psychological safety, our aim is to lean on key factors already discussed within broader established research to outline for the first time how these intersecting elements likely contribute to mental health in elite sport.

## What is Psychological Safety?

From its conception, the construct of ‘psychological safety’ was not developed, rationalised or understood in relation to competitive sport [18]. Thus, any application of psychological safety in the context of sport is, by its very nature, altered from the original intention. While the term has been in use since the 1960s [19], it was Kahn [20] who amplified interest in the area, defining psychological safety as “feeling able to show and employ one’s self without fear of negative consequences to self-image, status, or career” (p. 708) [20]. The term is now most frequently attributed to the significant work of Edmondson and colleagues [21, 22], who recently described psychological safety as “a belief that neither the formal nor informal consequences of interpersonal risks, like asking for help or admitting a failure, will be punitive” (p. 15) [23]. Also influential in this space, Clark [24] argues for a staged approach to psychological safety, comprising conditions in which individuals sequentially feel safe to “(1) feel included, (2) to learn, (3) to contribute, and (4) to challenge the status quo—all without fear of being embarrassed, marginalized, or punished in some way” (p. 6) [24].

As demonstrated by these interpretations, psychological safety as characterised in the organisational literature emphasises factors that promote an ability to learn (asking for help and allowing for mistakes along the way), to speak openly (particularly in contexts where power imbalances exist), and to challenge ideas, all without fear of negative interpersonal consequences. Typically, this is considered to benefit innovation, leadership and high performance (often measured via an organisational or common goal). Somewhat surprisingly, in the organisational literature, mental health has not been meaningfully featured as a part of, or significant consequence to, psychological safety [18].

In recent years, research and practice in sport has borrowed from this literature to investigate how psychological safety can be applied in sports contexts [11, 25–27]. However, in the transition from organisational psychology to elite sport, the term has become increasingly opaque, and in contrast to the organisational literature, is often discussed in the context of mental health [11–14]. We intuit that this likely reflects both the implied broader elements of the word ‘psychological*’* as incorporating well-being and mental health, and the intense research focus in recent years on mental health in elite sport [28]. Clarification is needed, however, as to whether—and if so, how—psychological safety is related to mental health in elite sport.

Advancing definitional clarity related to psychological safety in sport (recreational and elite), a recent systematic review summarised the broad literature conducted thus far [11]. The review identified 67 articles, yet concerningly, only 30% of these provided a specific definition of what was meant by the term. This highlights that when sports researchers and practitioners talk about psychological safety, they are likely referring to disparate ideas. Key definitional ingredients identified in the review were broad, and included the promotion of risk-taking, an absence of threat or harm, positive interpersonal relationships, positive emotional states, inclusivity, equality and respect [11]. This reinforces the importance of resolving definitional ambiguity and further introduces factors more closely aligned with well-being than those previously described in the organisational literature [18]. In trying to provide some kind of consolidation of this broad literature, Vella and colleagues proposed a sports-specific working definition of psychological safety as “the perception that one is protected from, or unlikely to be at risk of, psychological harm in sport” (p. 15) [11]. While this safeguarding from psychological harm is certainly a critical and fundamental ingredient, this definition might not sufficiently recognise other strategies and approaches to supporting mental health. Indeed, we suggest that psychologically safe environments should not only protect against psychological harm, but actively promote psychological well-being.

## The Unique Nature of Elite Sport and the Case for Mental Health Promotion

Elite sport is a unique context. For example, the pressures and decisions associated with risk and performance are typically played out in the public eye and in real time. In addition, elements in these ‘cultures’ rapidly change as athletes and staff rotate through an environment following changes such as deselection, resignations and injury. Thus, psychological safety—as originally conceived—does not apply as clearly as in traditional organisational environments. Indeed, this unique nature of elite sport may explain why explicit or implied deviations from earlier definitions of psychological safety are increasingly common [11]. Speaking to this concern, Taylor and colleagues critiqued the current applicability of psychological safety in elite sport more broadly [10], describing three issues in the field: (1) a lack of conceptual clarity, (2) a potential lack of transferability and (3) minimal consideration of potential negative consequences. These authors argue that with elite sport featuring such clear markers of success or failure/loss, and the subsequent consequences associated, assured safety from judgement is potentially unrealistic [10].

Acknowledging these realities of elite sport, role-related consequences (e.g. deselection) are likely when performance expectations are not met. Adding further complexity, difficult issues relating to mistakes, team selection, and performance outcomes among other factors in elite sport are commonly public, following media scrutiny and commentary. Thus, nuance is needed to better understand how psychological safety can be applied in the face of these realities and how to balance the risks to psychological safety that are inherent in the elite sport environment. Relational responses (e.g. social judgment, rejection) in the face of risk-taking (e.g. admitting mistakes, asking for help) are critical. However, how these factors may contribute to outcomes relating to mental health requires further exploration.

Elite sport environments are characterised by power differentials that can limit individuals from speaking up and disclosing potentially sensitive information. This may inhibit critically oriented performance-related conversations (i.e. questioning a coach’s strategy), as well as open dialogue about mental health (speaking with a club psychologist), both of which may contribute to negative consequences. Reducing hesitation to seek help and show one’s authentic and vulnerable self is a key component of psychological safety [18]. However, while previously this approach to help-seeking was based on pursuing learning and development, we argue that it has specific alignment with mental health help-seeking. It is therefore appropriate to target self-stigma that individuals in sport experience by disclosing and seeking support for their mental health [29].

It has been repeatedly shown that athletes in high performance contexts are fearful that speaking openly about a mental health problem will lead to negative consequences, including deselection or reduced perceptions of competence or ‘mental toughness’ [30–33]. We acknowledge that mental toughness (at least, as conceptualised by academics and practitioners) may well be critical to success in elite sport and is not necessarily in opposition to concepts congruent with good mental health [34, 35]. However, it is undoubtedly clear that many individuals in elite sport perceive that to disclose their mental ill-health could signal weakness or reduced ‘mental toughness’ to others in positions of influence (e.g. coaches) [30–33]. This is impactful, because when individuals feel a need to hide their mental health problems, they may be less likely to receive adequate professional support. Given this, in striving to achieve psychological safety, there must be a commitment to normalising and supporting the mental health needs of all athletes and staff within elite sports environments. While improvements in supporting athletes are evident in the literature and public discourse, there appears to have been less progress for coaches, support staff and administrators in these environments [5, 6, 36].

Our perspective is consistent with others implying that psychological safety is aligned with the pursuit of mental health in sport through its role in stigma and help-seeking. Henriksen et al. [12] describe that any initiative aimed at supporting athlete mental health through Olympic and Paralympic cycles must first and foremost improve the psychological safety of these high-performance environments. The 2021 IOC Mental Health Toolkit for Elite Athletes goes one step further, describing psychologically safe environments as those in which “athletes are comfortable being themselves, can take necessary interpersonal risks, have the knowledge and understanding of mental health symptoms and disorders, and feel supported and comfortable in seeking help if needed” (pp 34) [13]. This definition also incorporates aspects of mental health literacy, a critical underlying aspect that is necessary for individuals to recognise a need to both seek help and support others. To facilitate measurement of this in elite sport (and change in practices over time), several authors of the current paper developed the Sports Psychological Safety Inventory [14]. This self-report tool is validated with elite athletes, coaches and high-performance staff and aims to identify an individual’s perceptions of their sports environment with regards to stigma towards mental ill-health and their capacity to identify and disclose mental health concerns to others within their sport. Validation of this tool found the ‘mentally healthy environment’ and ‘low self-stigma’ domains were associated with lower scores on athlete-specific forms of psychological distress [14]. If sports organisations reinforce and promote environments which support safety around disclosures and management of mental ill-health, individuals will likely report better engagement with clinical services and subsequently mental health outcomes.

We see an underlying universal theme that relates to the ability to safely show one’s authentic self, without unfair or abusive judgement and unjustified negative repercussion [18]. In line with recent approaches which focus on safety from harm [11], we contend that psychological safety is explicitly relevant in the context of promoting mental health help-seeking and stigma reduction narratives within elite sports environments. Below we propose a theoretically informed model to describe this process.

## A Theoretically Informed Model of Psychological Safety and Mental Health in Elite Sport

**Fig. 1:**
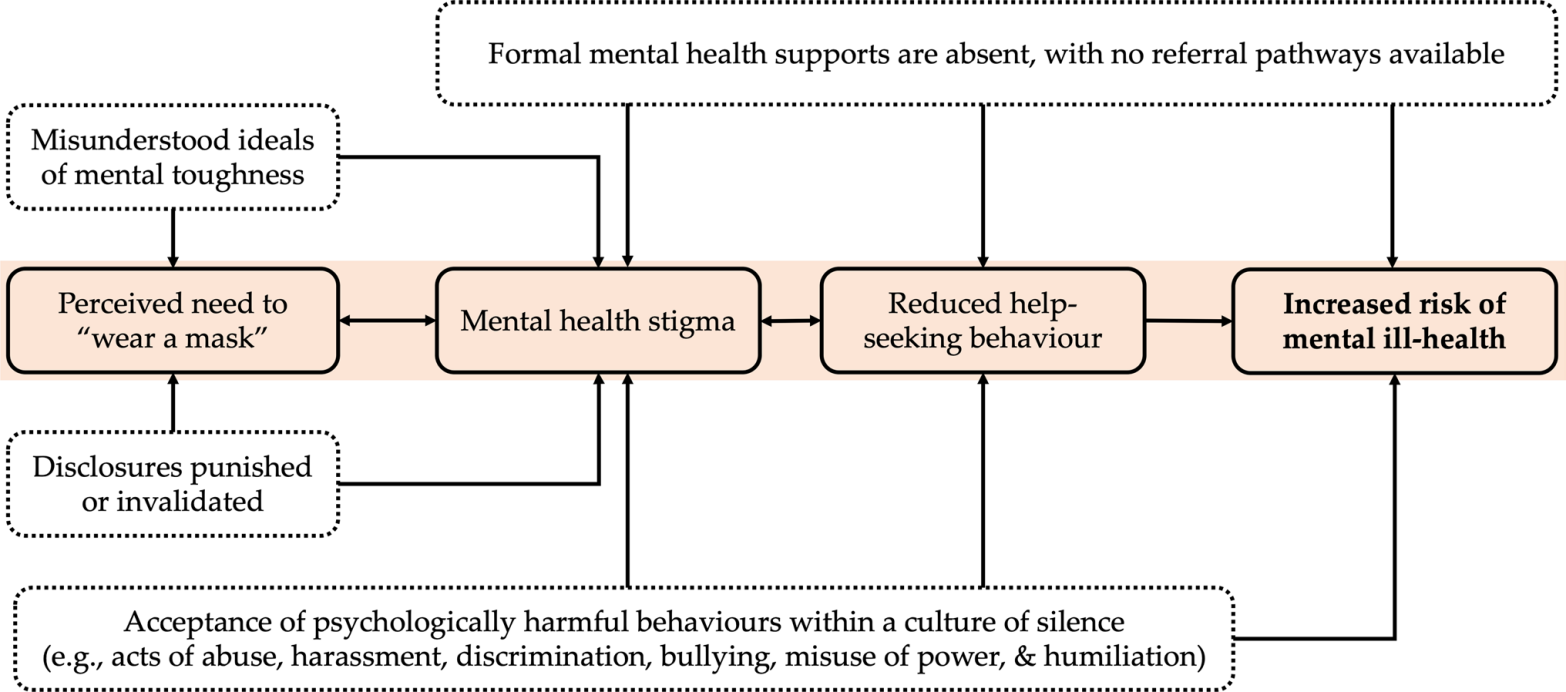
Representation of a psychologically unsafe environment. Boxes within the central red area represent the primary process in which psychological safety contributes to mental health, with checked boxes indicating key structural or cultural influencers

**Fig. 2:**
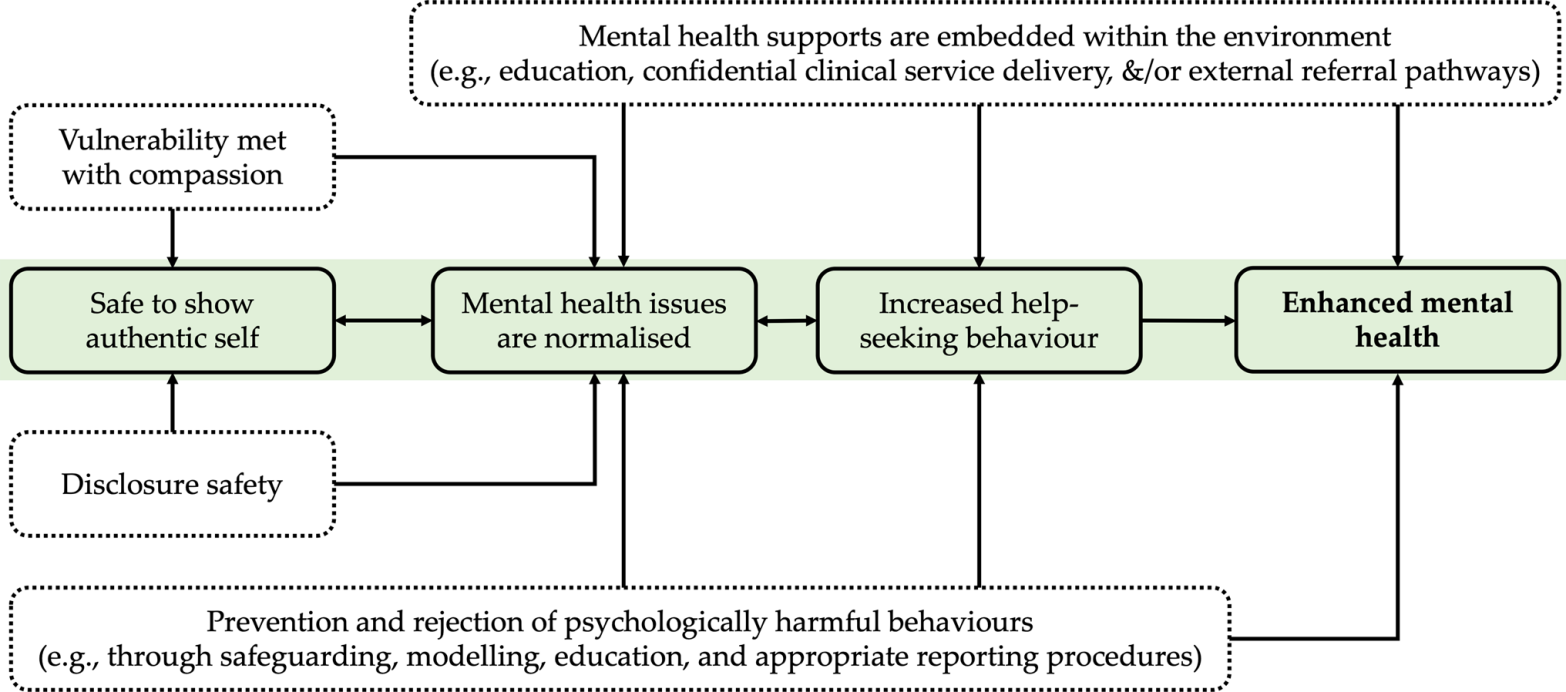
Representation of a psychologically safe environment. Boxes within the central green area represent the primary process in which psychological safety contributes to mental health, with checked boxes indicating key cultural influencers

To represent the role of psychological safety as a key contributor to enhanced mental health in elite sport, we propose a theoretical model informed by experience (clinical and applied), and the extant literature. Specifically, we outline two opposing environments to illustrate the effects of psychological safety (when absent or present) on mental health. First, Fig. 1 outlines how a psychologically unsafe environment could contribute to individuals being exposed to an increased risk of mental ill-health. In Fig. 2, we then represent how a psychologically safe environment can facilitate enhanced mental health.

### Psychologically Unsafe Elite Sports Environments Contribute to Mental Ill-Health

We contend that individuals in psychologically unsafe elite sports environments feel that they must hide their authentic self, which is a precursor to subsequent stages of the model. For example, among a sample of elite athletes diagnosed with a psychiatric disorder, participants described ‘wearing a mask’ to avoid perceptions of weakness and vulnerability in a high-performance environment [33]. We argue that individuals perceive the need to conceal their true experience as a result of two inputs: (1) their environment reinforcing ideals such as mental toughness, hegemonic masculinity (which can be present among any gender), stoicism and self-sufficiency, which are incorrectly presented or interpreted as oppositional to mental ill-health [37, 38] and (2) a hesitancy to disclose a mental health problem (or symptoms) for fear of invalidation or punitive repercussion (e.g. non-selection or losing trust of coaches and peers) [30]. This inability to show one’s authentic self or the tendency to ‘wear a mask’ when living with mental ill-health both contributes to, and is influenced by, all forms of mental health stigma.

Mental health stigma contributes to several important consequences in sport settings. Primarily, stigmatising environments lead to reduced help-seeking within the organisation [39]. Athletes have described how perceived stigma prevents them from reaching out within their sport for structured support, and instead leaning on loved ones and a trusted ‘inner circle’ for support [32]. A systematic review of factors impacting mental health treatment among elite athletes identified stigma as the strongest barrier to help-seeking [29]. Thus, if individuals in elite sport believe that they cannot reach out for support or referral, the nature of their mental health problems is likely to be maintained, and even exacerbated over time.

The model also accounts for psychological harms evident in sporting environments, as described by Vella et al. [11]. Psychological harms include, but are not limited to, acts of humiliation, rejection, sexual harassment, broken confidentiality and forms of discrimination according to sexuality, gender, ethnicity or physical status. A psychologically unsafe environment is one in which psychological harms are normalised and accepted in a culture of silence. Unfortunately, both physical and psychological harms are known to exist in elite sports environments [1], and high-profile cases have demonstrated how abusive practices within elite sports contexts can be tolerated or ignored in pursuit of success [40]. Furthermore, athletes have described that psychological harms, including coach maltreatment or negative comments relating to body image, can lead to increased mental ill-health [38]. Finally, we contend that if clear confidential pathways to mental health support and/or referral do not exist, then an environment becomes more psychologically unsafe.

### Enhancing Psychological Safety in Elite Sports Environments to Promote Mental Health

In Fig. 2, we propose the features that underpin a psychologically safe environment which may contribute to enhanced mental health outcomes. Thus, the relationships displayed here provide the preferred and sustainable alternative in contrast to the unsafe environment represented in Fig. 1. In promoting psychological safety, we contend that sports teams and organisations should have an explicit and direct focus on normalising and de-stigmatising experiences of mental ill-health, while concurrently reducing psychological harms within the environment.

First, individuals feel safe to show their authentic self (i.e. not needing a mask). This aligns with the concept of voice [23], recently described by Jowett and colleagues as when athletes are “empowered to take interpersonal risks (e.g., make mistakes, bring up problems, ask tough questions, embrace diversity) without fear of humiliation or retaliation by their peers” [p. 6., 25]. This is supported via multiple pathways. First, cultural norms around vulnerability and openness are reinforced throughout the organisation and its leaders [41]. For example, vulnerability around mental health should be met with compassion and support [42]. It has been shown that the attitudes and language of leaders (especially coaches) significantly influence how open athletes feel they can be about their own mental health [38]. Second, there must be clear evidence that individuals will not be punished for a mental ill-health disclosure (i.e. what we term disclosure safety). This can be done through clear policy and procedure, and culturally via awareness of previous athlete disclosures being well managed. In reinforcing these via organisational and cultural norms, it is anticipated that individuals will experience a sense of safety to show their authentic self. This leads to an environment where help-seeking behaviour is more likely to exist, and subsequently mental health issues are likely to be better managed, or potentially less present.

In addition, two broader organisational influences contribute throughout this pathway. First, where possible (i.e. in adequately funded professional or elite programs), there should be processes for easy access to mental health treatment and support either within the environment or via clear, confidential and accessible referral pathways [7, 43]; for example, see case studies in Canada [44] and Australia [45]. Second, organisations must take action to remove forms of psychological harm, through both proactive and reactive steps. This can be accomplished by establishing preventative and educational measures, reinforcing cultural norms of respect, diversity and inclusion, and by taking action to respond appropriately to known harmful events or individuals. These two approaches are foundational to influencing psychological safety in elite sports environments and likely contribute to normalisation and acceptance of mental health issues as a human experience, increased help-seeking and reduced causative or exacerbating risks.

### Psychological Safety is Maintained in Both Horizontal and Hierarchical Directions

An important consideration of this model is that actions aligned with psychologically safe environments are both maintained by, and benefit, all individuals across the entire sporting ecosystem [7, 8]. We emphasise that all individuals can contribute to psychologically safe (and unsafe) environments by their words and actions (e.g. contemptuous or mocking behaviours versus compassion for a teammate seeking psychological support). However, differing power distributions and hierarchies exist throughout elite sport and thus for an environment to truly embed psychological safety, these behaviours must start at the top of the organisation. Thus, leaders must be seen to actively promote and reinforce norms, actions and policies which support psychological safety. This is of special importance in elite youth settings where these power imbalances are even more prominent [16, 46]. Furthermore, to the responsibilities for implementation, psychologically safe elite sports environments must support all individuals within them, including athletes, coaches, executives and other support staff. Too often in the context of elite sports, the mental health needs of non-athletes are forgotten.

## Back to the Roots: Keeping Performance and Innovation in Mind

In this paper, we have explicitly focused on the role of psychological safety in mental health. However, we emphasise that the underlying ingredients described so far as essential to psychologically safe environments likely expand beyond goals associated with mental health. Embedded throughout our model are concepts of autonomy, authenticity and open, honest communication, all of which are important for supporting innovation, team cohesion and effectiveness. We align these principles with critical ingredients as originally described by leaders such as Kahn [20], Edmondson [22] and Clark [24]. Thus, while these core tenets of psychological safety support mental health, they are also likely essential elements of sustained peak performance. This is because avoidance of risk-taking for fear of making mistakes in a psychologically unsafe environment will limit the full range of performance capacity being achieved. Rather, in a psychologically safe environment where individuals feel safe to take risks in pursuit of sport-specific development, they may be better placed to enhance their skills or respectfully challenge leaders or established approaches in search of new ideas and growth. While beyond the intended focus of this paper, future holistic definitions of psychological safety may need to take this into account.

## Conclusion

In this *Current Opinion*, we have summarised what we see as key evidence pertaining to the role of psychological safety in support of mental health in elite sport. We have introduced a model informed by both the research literature and our collective applied experiences with elite sports contexts. We do this in the spirit of ongoing refinement through research and conversation about how best to enact psychological safety. Importantly, our model is testable, and longitudinal work, in addition to in-depth qualitative and mixed methods studies, is now necessary to support or adapt the different processes we outline. Thus, we call for an increase in research exploring the role of psychological safety in elite sport contexts, and further refinement of the term to best meet the needs of all those within elite sport.

## Footnotes

1 See https://www.iso.org/standard/64283.html.
